# Supplementary material for: Critical Review for the Production of Antidiabetic Peptides by a Bibliometric Approach
Source: Nutrients. 2022 Oct 14;14(20):4275. doi: 10.3390/nu14204275 (PMC9607871; doi:10.3390/nu14204275)
Supplement: Supplementary file 1 [file nutrients-14-04275-s001.zip › Supplementary Table S1.pdf]

Supplementary Table S1. Selected studies without peptide sequence and their respective outcomes of interest.

Authors (year)	Source of protein	Peptide Sequence	Outcomes of interest
Han, et al., 2021	Oilseed and dairy protein hydrolysates	_____	Oilseed proteins could be considered as comparable sources of ACE and alpha-glucosidase inhibitory peptides, especially soybean.
Kong, et al., 2021	Walnut (Juglans regia L.)	Table 1A	The peptide fraction with lower molecular weight and higher basic amino acid residues possessed strong DPP-IV inhibitory activity. Nine novel effective bioactive peptides were identified in the fraction.
Mazloomi, et al., 2021	Orange seed	_____	Could be used as a health-promoting ingredient to help in the reduction of blood pressure and the regulation of diabetes.
Rivero-Pino, et al., 2021b	Tenebrio molitor	Table 3 and Table 4	Potential ingredients in functional foods intended for the regulation of diabetes.
Acquah, et al., 2020	Review	Table 2	Bioactive peptides could serve as important primary strategies for management and/or control of diabetes.
Akan, E., 2020	Camel milk and donkey milk	_____	Peptides showed better antidiabetic and antioxidant activity compared to whey-derived peptides.
Harnedy-Rothwell, et al., 2020	Boarfish (Capros aper)	Table 3	IPVDM, a potent DPP-IV inhibitory activity in the in vitro assay, also showed potent activity in a cell-based DPP-IV inhibitory assay.

Authors (year)	Source of protein	Peptide Sequence	Outcomes of interest
Karimi, et al., 2020	Corn germ protein	———	The fractions showed high radical scavenging and α-glucosidase inhibitory activity, and α-amylase inhibitory activity can be attributed to high levels of hydrophobic amino acids.
Kehinde and Sharma, 2020	Review	All tables	Several BHs and BPs have been isolated from dairy, meat, cereals and legumes;
Li, et al., 2020	Arthrospira Platensis (Spirulina)	———	Tryptic phycobiliproteins hydrolysate is a new source of peptides for the development of nutraceuticals or functional foods.
Megrous, et al., 2020	Casein Hydrolysates	———	Casein hydrolysates generated by metalloendopeptidase under selected hydrolysis conditions showed significant antidiabetic properties.
Mudgil, et al., 2020	Quinoa (Chenopodium quinoa Willd.)	Table 2	Effective inhibitory properties towards enzymatic biomarkers of diabetes (DPP-IV and AG) and hypertension (ACE).
Ohara, et al., 2020	Common bean (Phaseolus vulgaris L. cv Carioca)	———	Antidiabetic potential was evidenced by total inhibition of alpha-amylase activity and reduction of alpha-glucosidase activity by 34.73%.
Olagunju, et al., 2020	Pigeon pea (Cajanus cajan)	———	Potential ingredients to formulate antihypertensive and antidiabetic functional foods and nutraceuticals.
Patil, et al., 2020	Review	Table 2	Bioactive peptides inhibit enzymes such as alpha-glucosidase, alpha-amylase, dipeptidyl peptidase-IV and glucose transporter systems involved in type 2 diabetes.

Authors (year)	Source of protein	Peptide Sequence	Outcomes of interest
Rivero-Pino, et al., 2020a	Sardine pilchardus	Table 3	The most bioactive fraction ranges from 800 to 1400 Da.
Rivero-Pino, et al., 2020b	Review	Table 2	The potential of bioactive peptides as antidiabetic agents to be employed in food formulation is a relevant field of research.
Rivero-Pino, et al., 2020c	Tenebrio molitor	——	Ultrasound pre-treatment modifies the native structure of the protein and subtilisin hydrolysis reduces the size of the peptide chain.
Wu, et al., 2020	Review	Review of peptides from cereals and pseudo cereals	Protein hydrolysates and peptides isolated from rice, wheat, oats, buckwheat, quinoa, barley, and corn have antidiabetic effects.
Yap, et al., 2020	Review	Table 1	Bioinformatics approach emerges as an innovative breakthrough to ameliorate the time and economic viability of traditional.
Zamudio and Campos, 2020	Review	Review of proteins and peptides from animal and plant sources focused on amaranth, quinoa and chia	Excellent alternative for further development of antidiabetic functional food and nutraceuticals.
Casanova-Martí, et al., 2019	Chicken feet (Gallus gallus domesticus)	________	Was a good source of DPP-IV inhibitors reducing glycaemia in glucose-intolerant rats and provided good stimulation of endogenous GLP-1 secretion.
Cermeño, et al., 2019	Porphyra dioica	Table 4	Peptides therein may be used as multifunctional ingredients in nutraceutical or functional food products.

Authors (year)	Source of protein	Peptide Sequence	Outcomes of interest
Connolly, et al., 2019	Brewers' spent grain	———	The extraction of bioactive peptides from wet BSG by direct hydrolysis is a viable method for processing BSG.
Gomez, et al., 2019	Portuguese Oyster (Crassostrea angulata)	———	Good source of peptides with ACE and DPP-IV inhibitory activities.
Ibrahim, et al., 2019	In silico analysis	Table 2	Potato-derived BP: antidiabetic and antimicrobial potentials; yam-derived BP: antihypertensive and anticancer agents.
Kęska, et al., 2019	Porcine (Sus scrofa) skeletal muscle	Table 4	Digested *in silico* by gastrointestinal enzymes have a high potential for the management of blood glucose levels in patients with T2DM.
Lima, et al., 2019	Chicken by-product	Table 2	Can potentially serve as ingredients of multi-functional foods with dual effects of DPP-IV inhibition and enhancement of cellular glucose uptake.
Liu, et al., 2019	Review	Table 2	Diets rich in specific bioactive ingredients, including food protein-derived peptides, have potential application in the prevention and management of T2DM.
Mudgil, et al., 2019	Camel skin (Camelius dromedaries)	———	ACE, DPP-IV and pancreatic α-amylase inhibition by camel skin gelatin hydrolysates was reported for the first time.
Park and Yoon, 2019	Perilla (Perilla frutescens var. japonica Hara)	———	Peptides fractionated by UF were found to have various physiological functions: antioxidant, antidiabetic and antihypertensive activities.

Authors (year)	Source of protein	Peptide Sequence	Outcomes of interest
Valencia-Mejía, et al., 2019	Beans (Phaseolus vulgaris L.)	———	Hydrolysis of common beans was able to produce molecules with higher hypoglycemic and antihyperglycemic activities.
Yan, et al., 2019	Review	Table 1	Bioactive peptides, particularly from natural products, show high potential for application in the management and treatment of diabetes.
González-Montoya, et al., 2018	Soybean protein	Table 2	First report. Inhibition of DPP-IV, α-amylase and intestinal α-glucosidases with potential antidiabetic properties.
Hall, et al., 2018	Cricket (Gryllodes sigillatus)	———	Peptides displayed good ACE, DPP-IV inhibition, and antioxidant activity; bioactivity increased, in most cases, after simulated gastrointestinal digestion.
Harnedy, et al., 2018	Atlantic salmon (Salmo salar)	———	Hydrolysates/peptides with significant antidiabetic (insulin and GLP-1 secretory and DPP-IV inhibitory) activity in vitro.
Mudgil, et al., 2018	Camel milk (Camelius dromedaries)	———	Camel milk protein hydrolysates effectively inhibited DPP-IV, lipase and α-amylase.
Nongonierma, et al., 2018a	Cricket (Gryllodes sigillatus)	———	First time report. The CP contains endogenous enzymes which were able per se to hydrolyze *G. sigillatus* proteins and yield samples with DPP-IV inhibitory properties.
Wang, et al., 2018	Walnuts (Juglans mandshurica Maxim.)	———	*In vivo*: WHPs alleviated insulin resistance by increasing insulin secretion, and liver GK and glycogen levels as well as by decreasing fasting blood glucose level.

Authors (year)	Source of protein	Peptide Sequence	Outcomes of interest
Neves, et al., 2017	Atlantic salmon (Salmo salar)	Table 2	Peptides have biological activities, such as ACE and DPP-IV inhibition, and antioxidant capacity.
Nongonierma, et al., 2017a	Camel milk (Camelus dromedarius)	Table 2 and Table 4	The first time that camel milk proteins serve as an interesting source of DPP-IV inhibitory peptides by using an *in silico* analysis.
Nongonierma, et al., 2017b	Milk protein isolate	Table 3 and Table 4	Some of these sequences were previously reported to be DPP-IV inhibitors or to possess structural features of DPP-IV inhibitory peptides.
Nongonierma, et al., 2017c	Wheat gluten	Table 4	Hydrolysate contained short (di- and tri-) peptides previously identified as DPP-IV inhibitors, and several peptides possessing DPP-IV inhibitory features.
Nongonierma, et al., 2017d	Review	Table 1	Potent DPP-IV inhibitory peptides have been identified in several food protein-derived hydrolysates.
Nongonierma, et al., 2017e	Bovine milk protein	Table 3 and Table 4	Several known, potent DPP-IV inhibitory peptides were identified within the milk protein hydrolysates.
Xia, et al., 2017	Review	Table 2	Natural peptides derived from several kinds of marine organisms showed great potential to regulate glucose metabolism for insulin-resistant individuals.
Mojica and Meíja, 2016	Common beans (Phaseolus vulgaris L.)	Table 2	Alcalase protein fractions showed outstanding antidiabetic potential by inhibiting the targeted enzymes through hydrogen bonds, polar and hydrophobic interactions.

Authors (year)	Source of protein	Peptide Sequence	Outcomes of interest
Nongonierma, et al., 2016a	Casein hydrolysates	———	A multi-functional hydrolysate, H12 (pH 8.0, 40 ∘C and 5 h), yielded high DPP-IV inhibitory and antioxidant activities.
Siow and Gan, 2016	Cumin seeds (Cuminum Cyminum)	———	Antioxidant activity may turn them into potential ingredients of health-promoting or functional foods.
Uraipong & Zhao, 2016	Rice bran (cultivar Reiziq)	———	α-amylase, α-glucosidase and ACE-inhibition activities comparable in magnitude to acarbose.
