# Supplementary material for: Critical Review for the Production of Antidiabetic Peptides by a Bibliometric Approach
Source: Nutrients. 2022 Oct 14;14(20):4275. doi: 10.3390/nu14204275 (PMC9607871; doi:10.3390/nu14204275)
Supplement: Supplementary file 1 [file nutrients-14-04275-s001.zip › Supplementary Table S2.pdf]

Supplementary Table S2. Studies that were selected and evaluated in the bibliometric review

| Authors (year) | Country | Type of inhibition | Type of analysis | Type of document | Journal | Citations |
|---|---|---|---|---|---|---|
| Han et al. (2021) | United Kingdom | α-glucosidase, DPP-IV | *in vitro* | Original | Current Research in Food Science | 0 |
| Kong et al. (2021) | China | DPP-IV | *in vitro* | Original | Food Chemistry | 0 |
| Mazloomi et al. (2021) | Spain, Iran | α-glucosidase, α-amylase | *in vitro* | Original | Foods | 0 |
| Mudgil et al. (2021) | United Arab Emirates, Malaysia | α-amylase, α-glucosidase, DPP-IV | *in vitro, in silico* | Original | Food Chemistry | 2 |
| Rivero-Pino et al. (2021a) | Spain | DPP-IV | *in vitro, in silico* | Original | Food Chemistry | 0 |
| Rivero-Pino et al. (2021b) | Spain | DPP-IV, α-glucosidase | *in vitro, in silico* | Original | Food & Function | 1 |
| Acquah et al. (2020) | Canada, Australia, New Zealand | α-amylase, α-glucosidase | *in vitro and in vivo* | Review | Critical Reviews in Food Science and Nutrition | 1 |
| Akan (2020) | Turkey | α-glucosidase, DPP-IV | *in vitro* | Original | Journal of Food Science and Technology | 1 |
| Feng et al. (2020) | China | α-glucosidase | *in vitro* | Original | International Journal of Food Science and Technology | 1 |
| Gao et al. (2020) | China | DPP-IV | *in vitro* | Original | Molecules | 0 |
| Harnedy-Rothwell et al. (2020) | Ireland, United Kingdom | DPP-IV | *in vitro, in situ* | Original | Food Research International | 2 |
| Ibrahim et al. (2020) | South Africa, Nigeria | DPP-IV, α-glucosidase | *in vitro, in silico* | Original | International Journal of Peptide Research and Therapeutics | 0 |
| Jia et al. (2020) | China | DPP-IV | *in vitro* | Original | Food Chemistry | 6 |
| Jin et al. (2020) | China | DPP-IV | *in vitro* | Original | Food Research International | 2 |
| Karimi et al. (2020) | Iran | α-glucosidase, α-amylase, DPP-IV | *in vitro* | Original | Food Science and Nutrition | 6 |

| | | | | | | |
|---|---|---|---|---|---|---|
| Kehinde and Sharma (2020) | India | DPP-IV | - | Review | Critical Reviews in Food Science and Nutrition | 17 |
| Li et al. (2020) | Italy | DPP-IV | *in vitro* | Original | Nutrients | 2 |
| Megrous et al. (2020) | China | α-glucosidase, α-amylase | *in vitro* | Original | International Journal of Peptide Research and Therapeutics | 0 |
| Mudgil et al. (2020) | United Arab Emirates, Malaysia, Ireland | DPP-IV | *in vitro, in silico* | Original | Journal of Cereal Science | 1 |
| Ohara et al. (2020) | Brazil | α-amylase, α-glucosidase | *in vitro* | Original | Biocatalysts and Biotransformation | 5 |
| Olagunju et al. (2020) | Nigeria, Canada | α-amylase, α-glucosidase | *in vitro* | Original | Journal of Food Biochemistry | 0 |
| Patil et al. (2020) | India | α-glucosidase, α-amylase, DPP–IV | *in vitro, in vivo* | Review | International Journal of Peptide Research and Therapeutics | 9 |
| Rivero-Pino et al. (2020a) | Spain | DPP-IV | *in vitro, in silico* | Original | Food Chemistry | 42 |
| Rivero-Pino et al. (2020b) | Spain | -- | *in vitro, in silico* | Review | Foods | 7 |
| Rivero-Pino et al. (2020c) | Spain | α-glucosidase | *in vitro* | Original | Food and Bioproducts Processing | 2 |
| Wu et al. (2020) | China | -- | *in vitro and in vivo* | Review | E3S Web of Conferences | 0 |
| Yap et al. (2020) | Malaysia | DPP-IV, α-amylase, α-glucosidase | *in silico, in vivo* | Review | Trends in Food Science and Technology | 2 |
| Zamudio and Campos (2020) | Mexico | α-amylase, α-glucosidase, DPP-IV | *in vitro and in vivo* | Review | Critical Reviews in Food Science and Nutrition | 0 |
| Casanova-Martí et al. (2019) | Spain | DPP-IV | *in vitro, in vivo* | Original | Food & Function | 6 |
| Cermeño et al. (2019) | Ireland | DPP-IV | *in vitro* | Original | Food & Function | 5 |
| Connolly et al. (2019) | Ireland | DPP-IV | *in vitro* | Original | Food Research International | 18 |

| | | | | | | |
|---|---|---|---|---|---|---|
| Gomez et al. (2019) | Philippines, Taiwan | DPP-IV | *in vitro, in silico* | Original | International Journal of Molecular Sciences | 4 |
| Ibrahim et al. (2019) | South Africa, Nigeria | DPP-IV | *in silico* | Original | International Journal of Peptide Research and Therapeutics | 6 |
| Kęska et al. (2019) | Poland | DPP-IV | *in silico* | Original | Nutrients | 2 |
| Lima et al. (2019) | Denmark | DPP-IV | *in vitro* | Original | Food & Function | 2 |
| Liu et al. (2019) | China, USA | DPP-IV | *in silico and in vivo* | Review | International Journal of Molecular Sciences | 47 |
| Mudgil et al. (2019) | United Arab Emirates | α-amylase, DPP-IV | *in vitro* | Original | LWT | 10 |
| Nongonierma et al. (2019) | Ireland, United Arab Emirates | DPP-IV | *in silico and in silico* | Original | Food Chemistry | 4 |
| Park and Yoon (2019) | Korea | α-amylase, α-glucosidase | *in vitro* | Original | Czech Journal of Food Sciences | 6 |
| Valencia-Mejía et al. (2019) | Brazil, Mexico | α-amylase, α-glucosidase | *in vitro, in vivo* | Original | Food Research International | 5 |
| Vilcacundo et al. (2019) | Spain, Ecuador | DPP-IV, α-amylase | *in vitro* | Original | Journal of the Science of Food and Agriculture | 7 |
| Wang et al. (2019) | China, United Kingdom | α-glucosidase, DPP-IV | *in vitro* | Original | Food Science and Nutrition | 26 |
| Xu et al. (2019) | China | DPP-IV | *in vitro* | Original | Journal of Agricultural and Food Chemistry | 3 |
| Yan et al. (2019) | China | α-amylase, α-glucosidase, DPP-IV | *in vitro* | Review | International Journal of Food Science and Technology | 13 |
| Zheng et al. (2019) | China | DPP-IV | *in vitro* | Original | Journal of Agricultural and Food Chemistry | 1 |
| González-Montoya et al. (2018) | Mexico and Spain | DPP-IV, α-amylase, α-glucosidases | *in vitro* | Original | International Journal of Molecular Sciences | 31 |
| Hall et al. (2018) | USA | DPP-IV | *in vitro* | Original | Food Chemistry | 13 |

| | | | | | | |
|---|---|---|---|---|---|---|
| Harnedy et al. (2018) | Ireland | DPP-IV | *in vitro* | Original | Food Research International | 6 |
| Ibrahim et al. (2018) | South Africa, Nigeria | α-glucosidase, α-amylase | *in vitro, in silico* | Original | Biomedicine & Pharmacotherapy | 22 |
| Mudgil et al. (2018) | United Arab Emirates, Malaysia | DPP-IV, α-glucosidase | *in vitro* | Original | Food Chemistry | 57 |
| Mune et al. (2018) | Cameroon, Germany | DPP-IV | *in vitro, in silico* | Original | Food Chemistry | 26 |
| Nongonierma et al. (2018a) | Ireland | DPP-IV | *in vitro* | Original | Food & Function | 2 |
| Nongonierma et al. (2018b) | Ireland, United Arab Emirates | DPP-IV | *in vitro, in silico* | Original | Food Chemistry | 9 |
| Wang et al. (2018) | China | α-glucosidase | *in vitro, in situ, in vivo* | Original | Journal of Food Biochemistry | 14 |
| Ji et al. (2017a) | China | DPP-IV | *in vitro* | Original | Journal of Chromatography B | 6 |
| Ji et al. (2017b) | China | DPP-IV | *in vitro* | Original | Journal of Food Science | 1 |
| Liu et al. (2017) | China | DPP-IV | *in vitro, in silico* | Original | Molecules | 4 |
| Mojica et al. (2017) | USA, Mexico | DPP-IV and α-glucosidase | *in vitro* | Original | Journal of the Science of Food and Agriculture | 32 |
| Neves et al. (2017) | Ireland | DPP-IV | *in vitro* | Original | Food Chemistry | 17 |
| Nongonierma et al. (2017a) | Ireland, United Arab Emirates | DPP-IV | *in vitro, in silico* | Original | Journal of Functional Foods | 43 |
| Nongonierma et al. (2017b) | Ireland | DPP-IV | *in vitro* | Original | Food Chemistry | 4 |
| Nongonierma et al. (2017c) | Ireland | DPP-IV | *in vitro* | Original | Food & Function | 2 |
| Nongonierma and FitzGerald (2017d) | Ireland | DPP-IV | - | Review | Journal of Food Biochemistry | 39 |
| Nongonierma et al. (2017e) | Ireland | DPP-IV | *in vitro* | Original | Food Research International | 8 |
| Song et al. (2017) | China | DPP-IV | *in vitro* | Original | Journal of Dairy Science | 31 |

| | | | | | | |
|---|---|---|---|---|---|---|
| Taga et al. (2017) | Japan | DPP-IV | *in vitro* | Original | Bioscience, Biotechnology, and Biochemistry | 2 |
| Uraipong and Zhao (2017) | Australia | α-glucosidase | *in vitro* | Original | Journal of the Science of Food and Agriculture | 20 |
| Vilcacundo et al. (2017) | Spain, Ecuador | DPP-IV, α-amylase and α-glucosidase | *in vitro* | Original | Journal of Functional Foods | 67 |
| Xia et al. (2017) | China | DPP-IV, α-amylase, α-glucosidase | *in vitro, in silico, in vivo* | Review | Marine Drugs | 27 |
| Lammi et al. (2016) | Italy | DPP-IV | *in vitro, in silico* | Original | Journal of Agricultural and Food Chemistry | 9 |
| Mojica and Mejía (2016) | USA | DPP-IV, α-amylase and α-glucosidase | *in vitro, and in silico* | Original | Food & Function | 12 |
| Nongonierma et al. (2016a) | Ireland | DPP-IV | *in vitro* | Original | Journal of the Science of Food and Agriculture | 19 |
| Nongonierma et al. (2016b) | Ireland | DPP-IV | *in vitro, in silico* | Original | Food &Function | 4 |
| Siow and Gan (2016) | Malaysia | α-amylase | *in vitro* | Original | Journal of Food Biochemistry | 13 |
| Uraipong and Zhao (2016) | Australia | α-amylase and α-glucosidase | *in vitro* | Original | Journal of the Science of Food and Agriculture | 61 |
